# Enhancing Testing Laboratory Engagement in Plant DNA Barcoding through a Routine Workflow—A Case Study on Chinese Materia Medica (CMM)

**DOI:** 10.3390/plants11101317

**Published:** 2022-05-16

**Authors:** Wai-Yan Ha, Ka-Lok Wong, Wai-Yee Ma, Yuk-Yu Lau, Wing-Han Chan

**Affiliations:** Government Chinese Medicines Testing Institute, Chinese Medicine Regulatory Office, Department of Health, Hong Kong, China; dnaanalyst@dh.gov.hk (K.-L.W.); magrayee@gmail.com (W.-Y.M.); slt1_rnd1@dh.gov.hk (Y.-Y.L.); sc_gcmti@dh.gov.hk (W.-H.C.)

**Keywords:** DNA barcode, quality control plan, CMM, plant species, differentiation

## Abstract

Introduction of DNA standards into Pharmacopoeia in different parts of the world enables identification of herbal materials in a complementary manner. However, little has been discussed about the quality requirements for a testing laboratory to implement DNA barcoding methods for herbal materials, which has limited the test method to be developed as a routine service. To encourage the engagement of testing laboratory in application of DNA barcode, a practical workflow including the components of analytical run and the corresponding quality control plan was suggested and employed to address a real-life challenge faced by the differentiation of plant-derived Chinese Materia Medica (CMM), Herba Potentillae Chinensis (Wei ling Cai), Herba Potentillae Discoloris (Fan Bai Cai), Radix Pulsatillae (Bai Tou Weng), and Radix Arnebiae (Zi Cao), which share similar morphological characteristics and multiple species involved. The ITS2 barcode results indicated that there are significant differences among the four CMM, together with quality control plan data to ensure the measurement traceability and validity of test results.

## 1. Introduction

A comprehensive Chinese Materia Medica (CMM) identification relies on the integration of multi-disciplinary approaches to acquire evidence of plant species’ origin through literature support or analysis of genetic markers, macroscopic characteristics by organoleptic examination, microscopic features by microscopy, and quantitative and qualitative analysis of chemical markers. Identification of botanical origin is one of the key elements in CMM differentiation for the sake of ensuring efficacy and safety of CMM being used originated from proper medicinal plants [1]. Hence, genetic traceability of plant sources of CMM has become a crucial concern. Superior to widely used identification techniques, genetic analysis seems to provide much information to reveal plant origin of CMM disregard of morphological features.

In recent decades, using DNA techniques for species identification has been commonly applied for herbal materials, especially in morphologically confused CMM, CMM originating from multiple sources, CMM without characteristic chemical markers, CMM of animal origin, and moderately processed CNM, such as in slice or powder form [1,2,3,4,5]. From 2010 onwards, Chinese Pharmacopeia (CP) has adopted DNA tests as a complementary tool for authentication of Chinese medicines to enhance the ability of species identification [6]. British Pharmacopeia has also launched DNA barcoding as a tool for botanical identification of herbal drugs in the supplementary chapter since 2017 [7]. USP in December 2014 recognized DNA testing to become an official technology and utilized it as a complement to chromatographic, spectroscopic, and botanical (microscopic or macroscopic) procedures [8]. These Pharmacopoeias have recognized that the DNA sequencing technique, which reveals the nucleotide order of a specific DNA region, can infer species origin by comparing that with the nucleotide order generated from an organism of known identity.

In 2003, Paul Hebert and his colleagues demonstrated the feasibility of a mitochondrial gene, cytochrome c oxidase (COI), for phylogenetic study of invertebrates at species level [9]. In this way, he proposed a system, called DNA barcoding, that utilizes the profiles or one or few standardized and relatively short DNA regions, called DNA barcodes, to identify all species of life. The Barcode of Life Data System (BOLD) was launched in 2005, which is a DNA barcode library and online platform specifically designed for repository, exchange, and manipulation of DNA barcoding information worldwide [10]. For plant species, the Plant Working Group of the Consortium for the Barcode of Life (CBOL) recommended *rbcL* and *matK* as plant barcodes [11]. Besides, other plant loci were also found to have significant variability at species level. For example, ITS2 was employed as the major barcode for the identification of CMM [2].

Government Chinese Medicines Testing Institute (GCMTI) was set up in in 2017, which specializes in the testing of, and scientific research on, Chinese medicines with a view of setting internationally recognized reference standards for the safety, quality, and testing methods of Chinese medicines. Driven by the roles of GCMTI in elevating local testing standards through technology transfer and promoting the development of Chinese medicine testing, GCMTI has integrated the essential elements from the guidance and quality control schemes published by the authorities during the development of DNA barcoding protocols: the technical guidance published by CP as the blueprint of the method, and the quality control requirements by the Hong Kong Laboratory Accreditation Scheme (HOKLAS) [12] as the governance of the validity of test results. A practical DNA barcoding workflow together with a quality control plan was designed to promote the application of DNA barcoding in testing labs in a systematic manner for routine service. GCMTI continuously accumulates test methods and DNA information to serve as a traceable platform for the local testing laboratories and Chinese Medicines industry (https://www.cmro.gov.hk/html/eng/useful_information/gcmti/research/index.html) (accessed on 13 April 2021).

To demonstrate the practicability of developed protocol, a case study of differentiation of four CMMs by the DNA barcode method was reported, including Herba Potentillae Chinensis (Wei ling Cai), Herba Potentillae Discoloris (Fan Bai Cai), Radix Pulsatillae (Bai Tou Weng), and Radix Arnebiae (Zi Cao). The Chinese medicines’ industry has raised concerns on these four CMMs, as these involve multiple-origin and closely related species, which make it difficult to identify these CMMs definitely. Herba Potentillae Chinensis and Herba Potentillae Discoloris are the aerial parts of two closely related species, *Potentilla chinensis* Ser. and *Potentilla discolor* Bunge, respectively, while Radix Pulsatillae is the root part of plant *Pulsatilla chinensis* (Bge.) Regel. These three CMMs share similar morphological features and are often miscollected or misused [13,14]. Radix Arnebiae is the root of two closely related species, *Arnebia euchroma* (Royle) Johnst or *Arnebia guttata* Bunge, and it has two groups of known unofficial substitutes: *Potentilla chinensis* and *Onosma* species (Family Boraginaceae). The former is a likely cause of confusion by its similar Chinese name Bei Zi Cao, used in Hong Kong markets, while the latter is the cause of substitution by its cheaper value [15].

## 2. Results and Discussion

### 2.1. Differentiation of Herba Potentillae Chinensis, Herba Potentillae Discoloris, Radix Pulsatillae, Radix Arnebiae, and Other Closely Related Species

Reference DNA sequences for this study (RDS) were retrieved and documented in Table S1, consisting of the four CMMs and addition of four other related species (*Potentilla kleiniana* Wight & Arn., *Pulsatilla cernua* (Thunb.) Bercht. & J.Presl, *Pulsatilla turczaninovii* Krylov & Serg., and *Lithospermum erythrorhizon* Siebold & Zucc.). According to data sources [16], retrieved RDS is a major ITS2 haplotype that is a consensus contig from multiple specimens and dominantly observed sequence type. The interspecific percent identities were calculated and are shown in Table 1, which ranged from 41.77 to 98.17.

In order to reveal the practicability of the workflow, three types of samples were collected, analyzed, and compared to RDS: (i) 37 authentic CMMs and the corresponding voucher plant specimens of the four CMMs; (ii) 3 available related herbal reference materials; (iii) 2 unknown samples. Each sample was tested in duplicate for the analysis of the ITS2 sequence. Table 2 shows the percentage identities of these samples against RDS.

For Herba Potentillae Discoloris and their corresponding plant voucher specimens, it was found that all were correctly matched to the corresponding RDS with percent similarities 98.3–99.1%. For Radix Arnebiae and its corresponding plant voucher specimen *A. euchroma,* results showed that they matched to the RDS of *A. euchroma* with percent similarities 99.1–100%, while they were only 76.1–76.3% to the RDS of *A. guttata*. For Herba Potentillae Chinensis and its corresponding plant voucher specimen, it was observed that RD480-1, RD480-2, and RD580-1 had 15 variable sites leading to the decrease of percent similarity. Referring to data source of RDS, actually, there are 22 variable sites in *Potentilla chinensis* [14]. By further comparing the variable sites in RD480-1, RD480-2, and RD580-1 against those in RDS, it was concluded that these three samples were more closely related to *Potentilla chinensis* than to *P. discolor* or *P. kleiniana*. For *P. chinensis*, the generated ITS2 sequences from each sample were compared with the RDS of *P. chinensis*, *P. cernua,* and *P. turczaninovii*. After comparison, it was revealed that the identity between a particular sample and the RDS of *P. chinensis* was always slightly higher than that of *P. cernua* and *P. turczaninovii*. For example, the identities between RD476-1A and the RDS of *P. chinensis*, *P. cernua,* and *P. turczaninovii* were 98.9, 97.0, and 97.0%, respectively. When comparing percent similarities among RDS and the collected samples, it was found that the interspecific values were lower than those of intraspecific; therefore, these four CMMs could be differentiated from each other definitely. From Table 1, the ITS2 marker was able to differentiate not only species of the four easily confused CMM but also other closely related species under genus either *Arnebia* or *Potentilla*. ITS2 marker was able to give high interspecific distances to enhance correct identification.

Related herbal reference materials of *Potentilla viscosa* J. Don (NIFDC), Radix Arnebiae (NIFDC), and Radix Onosmatis (NIFDC) were also used to test in the workflow. Results showed that Radix Arnebiae (NIFDC) was assigned to be *A. euchroma* with a percent identity of 100%, and was differentiated from its adulterant Radix Onosmatis (NIFDC), which originated from *Onosma hookeri*. The similarity of *Potentilla viscosa* J. Don (NIFDC) to three *Potentilla* species was 90–93%, while it was only 45–54% to other RDS species, which indicated that it was close to the genus *Potentilla*.

Unknown 1 and 2 samples were claimed to be Herba Potentillae Chinensis and Herba Potentillae Discoloris, respectively. However, the percent similarities of their closest RDS were 96 and 79% only, which were lower than the values observed in the collected authentic samples. Therefore, they were clearly not from any members of focal species. By homology searching in NCBI GenBank (https://blast.ncbi.nlm.nih.gov, accessed on 20 January 2022), Unknown 1 and Unknown 2 were matched closely to *Potentilla conferta* (Accession no. KT985760) and *Potentilla lineata* (Accession no. KP875291) with 100% percent identity, respectively.

### 2.2. Quality Control Data Generated in This Study

The validity of analytical performance of each run was checked for conformance with the acceptance criteria to determine if results of analysis were acceptable. Detail of the QC check results are shown in Table 3. According to the reasonable handling capacity for operator to conduct DNA analysis, we defined that system controls should be performed for each batch of samples, or every 10 test samples, whichever was the less. The QC results were passed in each control point and fully met the acceptance criteria.

### 2.3. Workflow and Quality Control Plan of the Protocol

One of the missions of GCMTI is to empower the industry through transfer of technology to strengthen quality control of CMM products. Given the DNA testing standards introduced in pharmacopoeias in different parts of the world and the well-established testing service requirements set by the local accreditation body, GCMTI links up both elements to facilitate the engagement in DNA barcode technology for CMM identification of testing laboratory through the design of routine workflow, validated test protocols, and the continuous expansion of reference DNA sequence library (the Library) to meet the local need.

GCMTI has published a step-by-step test protocol which is open for assess [17]. The test protocols and the Library have several key features: (a) the selected barcodes are adopted in national Pharmacopeias or widely accepted by the scientific communities for the purpose of species identification; (b) the Library furnishes up to three DNA barcodes for each specimen to strengthen discrimination capability and flexibility; (c) specimens (including voucher specimens and authentic CMM) for the Library have good collection records with known provenance. They are mainly obtained from the curation of Hong Kong Chinese Materia Medica Standards (HKCMMS) program, which have been authenticated by experts in the field. The organoleptic and chemical characteristics of CMMs are well documented in the editions of HKCMMS, which enhances the genetic traceability between CMM and original species; (d) to ensure the quality and validity of reference DNA sequences, two independent measurements for each reference material would be conducted; (e) the method validation has been conducted in accordance with HOKLAS requirements with satisfactory results in an inter-laboratory comparison study, which makes the developed DNA barcode method have high transferability to the testing industry.

**Figure 1:**
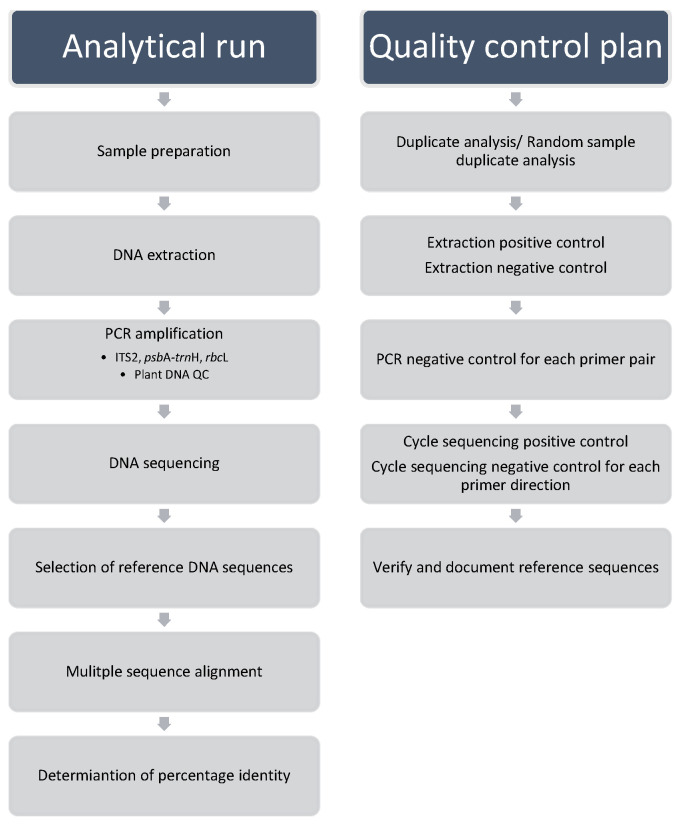
Workflow of DNA barcode test method and quality control plan. System controls should be run in parallel with each batch of test samples to govern the analytical performance of each run. In consideration of the compliance with QC plan, the operator should determine the number of samples in a batch to give a reasonable handling capacity to conduct DNA analysis.

In addition to the workflow and the Library, an effective quality control plan is essential for a testing laboratory to provide consistent testing service and maintain validity of test results. Figure 1 shows a schematic representation of established DNA barcoding workflow together with a quality control plan.

The analytical run includes the generation of DNA barcodes, selection of reference DNA sequences, data comparison by multiple sequence alignment, and determination of percentage identity for species discrimination. Three DNA barcodes were provided for selection to enhance species discrimination depending on the test scope to be provided by the testing laboratory. Following the quality control requirements recommended by local accreditation body, the analytical performance of each run was checked for conformance with the acceptance criteria to determine if results of analyses are acceptable and able to meet the objective of the DNA barcoding test method. System controls, performed for each batch of test samples, include sample duplicate or random sample duplicate controls to check for consistence of test result; extraction positive and negative controls to reveal the validity of reagents and the performance of the extraction protocol; PCR negative controls and cycle sequencing negative controls to demonstrate the absence of contaminating nucleic acids in the PCR reagent and cycle sequencing reagent; addition of plant DNA QC PCR system to check DNA extract being amplifiable; cycle sequencing positive control to demonstrate the ability of the cycle sequencing amplification and electrophoresis separation to determine the accurate base pair composition of the target amplicon.

### 2.4. Selection of DNA Barcodes

Selection of reference DNA sequences for data comparison is crucial for the reliability and validity of test results. By selecting appropriate DNA marker(s), it enables users to identify species by matching with reference DNA sequence of organisms with known provenance to reveal taxonomic identity or to differentiate target species of interest from group of organisms.

In this study, ITS2 was selected as the DNA barcodes for analysis. According to Chapter “9107 Guidelines for molecular DNA barcoding of Chinese Materia Medica” of Chinese Pharmacopoeia (CP), two DNA regions, i.e., ITS2 and *psbA*-*trnH*, were selected as DNA barcodes for land plants derived CMM. ITS2 is the major barcode and *psbA*-*trnH* is an auxiliary DNA barcode. As the targets of this work are Chinese Materia Medica and their related species, ITS2, instead of *matK* or *rbcL* as suggested by BOLD [10], was used. The major ITS2 haplotype is an optimal choice for DNA comparison that enables a simplified DNA alignment for distance analysis. However, for some species with high intra-species variation such as *Potentilla chinensis*, more individual data should be available to evaluate variable sites and determine intra- and inter-specific distance in order to make an accurate interpretation.

Other DNA barcodes for genetic identification plant species, such as members under the family of Ranunculaceae [18,19,20], Rosaceae [21], and Boraginaceae [22], have not been reported. With the availability of reference DNA sequences in the public databases or published dataset [16], the suggested workflow governed by certain quality control requirements can be easily employed for differentiation of members of focal species to address real-life challenges. Testing laboratories can retrieve and document DNA sequences for comparison in accordance with their service needs, which makes DNA barcoding flexible to expand testing scopes, given that reliable reference DNA sequences for particular species are available.

### 2.5. Further Points Taken into Consideration to DNA Barcoding

Reference DNA sequences built up from authentic specimens or certified reference materials obtained from reputable national institutes, authorities, or museums are highly reliable. Acquiring DNA data from public genetic sequence databases such as BOLD, Genbank, or RefSeq of National Center for Biotechnology Information (NCBI), as well as other published DNA libraries [16] as reference DNA sequences is also acceptable. However, selection criteria should be determined, properly verified, and documented in order to maintain measurement traceability. Reference DNA sequences to be used can be verified by one of the following means: (a) the sequences were independently submitted by two different groups of researchers and the identity of the two submissions is 100%; (b) the sequence was submitted by a recognized laboratory, which has published significant work on the target species/varieties [12]. In some circumstances, more reference DNA sequences from different individuals of the same species may be used to determine nucleotide variations to facilitate the interpretation of test result. The selected reference DNA sequences individually or made consensus contig (a set of overlapping DNA segments that together represent a consensus region of DNA) as major haplotype sequence can be used for homology matching with that of test samples by multiple sequence alignment and to obtain percent identity to infer the relatedness of test samples.

The DNA barcoding workflow in this study employed a direct sequencing technique to determine the nucleotide order of DNA barcodes. The sequencing technique is a gold standard, which is widely accepted by several recognized Pharmacopoeias as well as accreditation bodies for authentication, making it a good platform for testing laboratory to adopt for routine services. Such an approach enables testing laboratories to shorten turnaround times and labor intensive work from cloning. This method is particularly suitable for raw or moderately processed herbal material in whole or powdered form from the single species origin. However, it is not an optimal sequencing technique for mixed CMM samples, as unambiguous and overlapped peaks will be produced. Next-generation sequencing (NGS) is a better choice as it can determine a pool of mixed species of DNA fragments individually and simultaneously. However, a much higher cost of investment for testing laboratory and much more knowledge on bioinformatics may be required. Depending on the operation need, the testing laboratory may adopt NGS to this workflow. DNA barcode information generated in this study together with quality control for NGS can possibly be a tool for mixed CMM samples.

## 3. Materials and Methods

### 3.1. Collection of Materials

Voucher plant specimens and authentic CMM were collected from the GCMTI herbarium and Hong Kong Chinese Materia Medica Standards (HKCMMS) projects, respectively. Each plant specimen and medicinal material was authenticated by experts, well-documented, and deposited in GCMTI. Extraction positive control materials and herbal powdered materials were obtained from the National Institutes for Food and Drug Control (NIFDC).

### 3.2. DNA Barcode Test Method and Quality Control Plan

The workflow in parallel with quality control plan is depicted in Figure 1. The generic protocol covered (1) sample preparation, (2) DNA extraction by manual operation or automated DNA workstation and evaluation of DNA quality and quantity, (3) polymerase chain reaction (PCR), PCR product check and cleanup, (4) Sanger DNA sequencing, and (5) post sequencing analysis supplemented with quality control parameters, which are openly published in the GCMTI website [17]. It has been validated on herbal powdered materials of various medicinal parts, mainly rhizoma, radix, stigma, cortex, fructus, herba, flos, semen, folium, stamen, plumula, and rhizomatis nodus obtained from NIFDC. Depending on the operational demand of a testing laboratory, the step-by-step protocol had the flexibility to conduct in a way of assistance of automation tools for medium to high throughput or by manual operation of low cost investment.

In some circumstances, minor modification of the generic protocol might be required to tackle the different natures of CMM, to enhance the efficiency of extraction and PCR. Radix Arnebiae and Herba Potentillae Discoloris, which were rich in PCR inhibitors, should have a pre-treatment step, to decrease adverse effects on downstream processes. The input materials were reduced to 25 mg and exposed to additional washing steps with nuclease-free water four times before adding extraction buffer. The eluted DNA was further cleaned up with DNA Clean & Concentrator-5 kit (Zymo Reserach) in accordance with the instruction manual. PCR cycling conditions are given in the published generic protocol [17].

### 3.3. Data Analysis and Species Discrimination

CodonCode Aligner version 9.0.1 was used to process sequence files into a usable unit, a consensus sequence, for NCBI BLAST analysis or for comparison with sequences from a database of authentic reference materials. The analytical steps involved were sequence assembly, sequence alignments, sequence editing and end clipping. For ITS2 sequences, the consensus sequences were further subjected to annotation using a tool at http://its2.bioapps.biozentrum.uni-wuerzburg.de/ (accessed on 16 November 2020). The data quality of sequencing controls were reviewed by Sequence Scanner software version 2.0. The cycle sequencing positive control, pGEM, should give the minimum CRL in the analyzed sequence at least 650 bp with trace scores ≥20. The cycle sequencing negative control for all primers should give raw data without signal, which was observed as a flat signal profile without or with a sharp peak of unincorporated dyes at early scan number in raw data. To differentiate multiple species, the nucleotide sequences obtained from test samples and reference materials of NIFDC were further compared with the major ITS2 haplotype published by Chen SL [16]. Multiple sequences alignment was conducted using MUSCLE and the percent identity based on Tamura-Nei distance model was determined using Geneious Prime 2019.0.3 (https://www.geneious.com/) (accessed on 30 March 2021).

## 4. Conclusions

With the accumulation of DNA barcode information deposited in data repositories, DNA barcoding is now one of the key identification tools for organisms, which can complement traditional morphological approaches. Plenty of studies have concluded that it could resolve phylogenetic relationships of a variety of species. To bring and link these research outcomes into real-life application for testing industries, a quality control plan plays an essential role for monitoring critical factors in the whole analytical process to produce reliable and reproducible results. In the present study, we demonstrated the applicability of a validated DNA barcoding workflow with proper quality control plan for differentiation of four plant-derived CMMs. In our analyses, ITS2 barcode was able to distinguish not only species of the four easily confused CMMs but also other closely related species. Interspecific distances among species were higher than those of intraspecific which enhance correct identification. The validity of analytical performance of each run was checked for conformance with the acceptance criteria to determine if results of analysis were acceptable.

## Figures and Tables

**Table 1:** Percent identity of reference DNA sequence for this study (RDS) [14].

Reference	*Arnebia euchroma *	*Arnebia guttata *	*Pulsatilla cernua *	*Pulsatilla chinensis *	*Pulsatilla turczaninovii *	*Potentilla chinensis *	*Potentilla discolor *	*Potentilla kleiniana *
*Lithospermum erythrorhizon*	82.89	80.27	41.77	42.46	42.06	48.95	49.37	48.96
*Arnebia euchroma*		85.33	43.15	43.43	43.82	48.95	48.1	48.54
*Arnebia guttata*			43.32	43.2	43.6	46.84	46.41	46.03
*Pulsatilla cernua*				98.15	98.15	57.96	56.64	56.58
*Pulsatilla chinensis*					98.17	57.71	56.39	56.77
*Pulsatilla turczaninovii*						58.15	56.83	56.77
*Potentilla chinensis*							92.86	91.98
*Potentilla discolor*								92.92

**Table 2:** DNA barcode ITS2 test results for CMM and the corresponding plant voucher specimens. The percentage identity was calculated using Geneious Prime.

			Percent Identity with ITS2 (%)								
CMM/Plant Specimen	Locality	Sample No.	*Potentilla discolor*	*Potentilla chinensis*	*Potentilla kleiniana*	*Pulsatilla cernua*	*Pulsatilla chinensis*	*Pulsatilla turczaninovii*	*Arnebia euchroma*	*Arnebia guttata*	*Lithospermum erythrorhizon*
**Radix Pulsatillae**	Lushi, Henan	RD476-1	55.11	56.44	55.95	96.99	98.85	97.03	42.03	40.60	38.84
	Lushi, Henan	RD476-3	55.56	56.89	56.39	97.45	99.31	97.95	42.43	41.00	39.44
	Lushi, Henan	RD476-4	55.56	56.89	56.39	97.45	99.31	97.95	42.43	41.00	39.44
	Tonghua, Jilin	RD477-1	55.11	56.44	55.95	97.22	99.08	97.26	42.23	40.80	39.04
	Tonghua, Jilin	RD477-2	55.11	56.44	55.95	97.22	99.08	97.26	42.23	40.80	39.04
	Fuping, Hebei	RD478-1	55.56	56.89	56.39	97.69	99.54	97.72	42.03	40.80	39.44
	Fuping, Hebei	RD478-2	55.11	56.44	55.95	97.22	99.08	97.26	42.23	40.80	39.04
**GB0000096a00**	Tonghua, Jilin	RD576-1	55.78	57.11	56.61	97.92	99.77	98.40	42.43	41.00	39.64
**GB0000098a00**	Fuping, Hebei	RD577-1	55.56	56.89	56.39	98.15	100.00	98.17	42.23	40.80	39.44
**Herba Potentillae Chinensis**	Hunan, Shimen	RD479-1	92.86	100.00	92.45	57.14	56.89	57.33	49.57	46.58	46.38
	Hunan, Shimen	RD479-2	94.29	98.57	93.16	56.70	56.44	56.89	49.79	47.01	47.02
	Hunan, Shimen	RD479-3	93.10	99.76	92.69	57.14	56.89	57.33	49.36	46.79	46.60
	Yunnan, Qujing	RD480-1	96.19	96.67	93.16	56.47	56.22	56.67	49.57	46.79	47.45
	Yunnan, Qujing	RD480-2	95.95	96.43	92.92	56.70	56.44	56.89	49.36	46.58	47.23
***Potentilla chinensis* Ser.** **GB0000101a00**	Yunnan, Qujing	RD579-1	92.86	100.00	92.45	57.14	56.89	57.33	49.57	46.58	46.38
**GB0000102a00**	Yunnan, Qujing	RD580-1	96.19	96.67	93.16	56.47	56.22	56.67	49.57	46.79	47.45
**Herba Potentillae Discoloris**	Woyang, Bozhou, Anhui	RD484-1	98.33	92.38	92.69	55.13	54.89	55.33	49.36	46.79	48.30
	Woyang, Bozhou, Anhui	RD484-2	98.57	92.38	92.92	54.91	54.67	55.11	49.36	46.79	48.30
	Huoshan, Anhui	RD485-1	98.81	92.62	93.16	55.13	54.89	55.33	49.36	46.79	48.30
	Huoshan, Anhui	RD485-2	99.05	92.86	93.40	55.36	55.11	55.56	49.57	47.01	48.51
	Xiangyang, Hubei	RD486-1	99.05	92.86	93.40	55.36	55.11	55.56	49.57	47.01	48.51
	Xiangyang, Hubei	RD486-2	98.81	92.62	93.16	55.13	54.89	55.33	49.36	46.79	48.30
***Potentilla discolor* Bunge** **GB0000105a00**	Woyang, Bozhou, Anhui	RD582-1	98.33	92.38	92.69	55.13	54.89	55.33	49.15	46.58	48.09
**GB0000108a00**	Huoshan, Anhui	RD583-1	98.81	92.62	93.16	55.36	55.11	55.56	49.36	46.79	48.30
**GB0000110a00**	Xiangyang, Hubei	RD584-1	99.05	92.86	93.40	55.36	55.11	55.56	49.57	47.01	48.51
**Radix Arnebiae**	Wulumuqi, Xinjiang	RD481-1	49.15	49.15	49.57	42.34	42.23	43.03	99.10	76.30	80.87
	Wulumuqi, Xinjiang	RD481-2	49.15	49.15	49.57	41.94	41.83	42.63	99.10	76.09	80.87
	Kashi, Xinjiang	RD482-1	49.15	49.15	49.57	41.94	41.83	42.63	99.10	76.09	80.87
	Kashi, Xinjiang	RD482-2	49.15	49.15	49.57	41.94	41.83	42.63	99.10	76.09	80.87
	Akesu, Xinjiang	RD483-1	49.15	49.15	49.57	41.94	41.83	42.63	99.10	76.09	80.87
	Akesu, Xinjiang	RD483-2	49.15	49.15	49.57	41.94	41.83	42.63	99.10	76.09	80.87
***Arnebia euchroma* (Royle) Johnst** **GB0000111a00**	Wulumuqi, Xinjiang	RD587-1	49.57	49.57	50.00	42.34	42.23	43.03	100.00	76.09	80.87
**GB0000112a00**	Wulumuqi, Xinjiang	RD588-1	49.57	49.57	50.00	42.34	42.23	43.03	100.00	76.09	80.87
**GB0000113a00**	Kashi, Xinjiang	RD589-1	49.57	49.57	50.00	42.34	42.23	43.03	100.00	76.09	80.87
**GB0000114a00**	Kashi, Xinjiang	RD590-1	49.57	49.57	50.00	42.34	42.23	43.03	100.00	76.09	80.87
**GB0000115a00**	Akesu, Xinjiang	RD591-1	49.57	49.57	50.00	42.34	42.23	43.03	100.00	76.09	80.87
**GB0000116a00**	Akesu, Xinjiang	RD592-1	49.57	49.57	50.00	42.34	42.23	43.03	100.00	76.09	80.87
***Potentilla viscosa* J. Don (NIFDC)**	-	B-HT-334	91.67	93.06	90.37	54.78	54.55	54.98	46.25	45.00	45.23
**Radix Arnebiae (NIFDC)**	-	B-HT-327	49.57	49.57	50.00	42.34	42.23	43.03	100.00	76.09	80.87
**Radix Onosmatis (NIFDC)**	-	B-HT-335	49.15	48.31	48.94	38.80	39.29	39.53	77.51	69.65	75.99
**Claim to be** **Herba Potentillae Chinensis)**	-	Unknown 1	96.21	94.31	93.90	55.56	55.31	55.75	48.94	47.66	46.61
**Claim to be** **Herba Potentillae Discoloris)**	-	Unknown 2	78.60	77.67	78.34	50.22	50.87	50.00	47.70	45.96	45.00

**Table 3:** Evaluation of quality control results.

Control Point	Quality Control Parameters	Material Used	Acceptance Criteria	QC Results
Entire	Sample duplicate/Random sample duplicate	Each test sample	Consistent test results of duplicate samples across test procedure	Passed
DNA extraction	Extraction positive control	Control materials/reference materials	PCR positive result shown in each DNA barcode system	Passed
	Extraction negative control	Extraction reagent blank	PCR negative finding	Passed
DNA amplification	Plant DNA QC PCR	Each test sample	PCR positive result with amplicon of about 100 bp	Passed
	PCR negative control	PCR reagent blank	PCR negative finding	Passed
Cycle sequencing	Cycle sequencing positive control	pGEM	Cycle sequencing positive finding, i.e., minimum Contiguous Read Length (CRL) * at least 650 bp with trace scores ≥20	Passed
	Cycle sequencing negative control	Sequencing reagent blank	Cycle sequencing negative finding, i.e., raw data without signal	Passed
Choice of reference sequences	Verify reference sequence	In-house reference DNA sequence/public reference DNA sequences	HOKLAS requirement [12]	Passed and Documented

* The length of CRL can be varied depending on the amplicon size of DNA barcode marker and electrophoresis run module to be selected. In general, the CRL of cycle sequencing positive controls should be longer than the amplicon size of the DNA barcode to be analyzed.

## Data Availability

The sequences presented in this study are openly available in GenBank with reference number OM180086-OM180123.

## Supplementary Materials

The following supporting information can be downloaded at: https://www.mdpi.com/article/10.3390/plants11101317/s1, Table S1: Reference DNA sequences selected for the study of easily confused CMM. References [16,23,24] are cited in the supplementary materials.
