# Supplementary material for: Enhancing Testing Laboratory Engagement in Plant DNA Barcoding through a Routine Workflow—A Case Study on Chinese Materia Medica (CMM)
Source: Plants (Basel). 2022 May 16;11(10):1317. doi: 10.3390/plants11101317 (PMC9146924; doi:10.3390/plants11101317)
Supplement: Supplementary file 1 [file plants-11-01317-s001.zip › plants-1572290-supplementary.pdf]

Table S1. Reference DNA sequences selected for the study of CMM.

CMM	Plant species	Sequence source	Major ITS2 haplotype	Amplicon (bp)	Variation sites	Reference
**Herba Potentillae Chinensis**	*Potentilla chinensis* Ser.	17 CMM collected from 6 locality, 2 plant specimens from 2 locality, 1 NIFDC reference material, 3 counterchecking specimens	CACGTCGTTGCCCCTCCCAACCCCTTCGGGAGTTGGCTGGGACGGATGATGGCCTCCCGTGTGCTCCGCCGCGCGGTTGGCATAAATAACGAGTCATCGGCGGCCAACGCCGCGACAATCGGTGGTTGTCAAACCTCGGTGTCCTGTCGCGCGCGCGTCGTCCTCGGCTCTTCCGATCTAATGCGCGTCGGTTCGCCGGCGCTTTCAACG	212	22 sites: T26C, T62C, T184C, T195C, A49G, A182G, C64G, C165G, C66T, C69T, C152T, C163T, C171T, C175T, C198T, G91A, G177A, G201A, A96C, C156A, T164G, G193M, in/del 169, 174	[1]
**Herba Potentillae Discoloris**	*Potentilla discolor* Bunge	7 CMM from 2 locality, 1 plant specimen, 1 NIFDC reference	CACGTCGTTGCCCCTCCCAACCCCTCCGGGAGTTGGGTGGGACGGATGATGGCCTCCCGTGCGCTCCGTCGCGCGGTTGGCATAAATAACAAGTCCTCGGCGGCCAACGCCGCGACAATCGGTGGTTGTCAAACC	210	3 sites: C37G, G93T, T163C	[1]

		material, 2 counterchecking specimens	TCGGTGTCCTGTCGCGTGCGAGTCGTCCGGGGCTTTTCCAATCTGATGCGCGTCGATTCGTCGGCGCTTTCAACG			
**-**	*Potentilla kleiniana* Wight et Arn.	Plant specimens	CACGTCGTTGCCCCTCCCAACCCCTTCGGGAGTTGGCTGGGACGGATGGTGGCCTCCCGTGTGCTCCGTCGCGCGGTTGGCATAAATAACAAGTCCTCGGCGGCCAACGCCGCGACAATCGGTGGTTGTCAAACCTCGGTGTCCTGTCGCGCGCGCGTCGTCCGGGGCTTTTTTTCAATCTAACGCGCGTCGCTCCGTCGACGCTTTCAACG	212	-	[1]
**Radix Pulsatillae**	*Pulsatilla chinensis* (Bge.) Regel	13 CMM collected from 4 locality, 14 plant specimen collected from 3 locality, 1 NIFDC reference	CACACAGCGTCGCCCCCACCAAAGCATTTGGATGGGGGCGGAAATTGGCCCCCCGAGCCCCCCGGGCACGGTCGGCACAAATGTTGGCCCTCGGCGGCGAGCGTCGCGGTCAGCGGTGGTTGTACTCTCATCCTCCAAAGACAAAATGACGCGTCCGCCTCGTCGCCCGCTGGGCGA	219	8 sites:<br>C17T, C61T, C63T, C112T, C126G, G170A, G193T, A201T, in/del 64	[1,2]

		material, 2 counterchecking specimens, Accession no. GU732650	AGATGACCCAAGGAGTCTCCCC AACCGGAGACTTCCACCTG			
**Radix Pulsatillae Cernuae**	*Pulsatilla cernua* (Thunb.) Bercht.et Opiz.	Accession JN811070 & HQ829820	ACAGCGTCGCCCCCACCAAAGC ATTTGGATGGGGGCGGAAATTG GCCCCCCGAGCCCCCCGGGGCA CGGTCGGCACAAATGTTGGCCC TCGGCGGCGAGCGTCGCGGTTA GCGGTGGTTGTACTCTCATCCTC CAAAGACAAAATGACGCGTCCG CCTCGTCGCCCGCTGGGCAAAG ATGACCCAAGGAGTCTCCCCTA CCGGAGACTTCCACCTG	216	-	[1]
**-**	*Pulsatilla turczaninovii* Kryl. Et Serg.	Accession GU732649	CACACAGCGTCGCCCCCACCAA AGCATTTGGATGAGGGCGGAAA TTGGCCCCCCGAGCCCTCCGGG GCACGGTCGGCACAAATGTTGG CCCTCGGCGGCGAGCGTCGCGG TCAGCGGTGGTTGTACTCTCATC CTCCAAAGACAAAATGACGCGT	219	-	[1]

			CCGCCTCGTCGCCCGCTGGGCG AAGATGACCCAAGGAGTCTCCC CTACCGGAGACTTCCACCTG			
**Radix Arnebiae**	*Arnebia euchroma* (Royle) Johnst	11 CMM collected from 6 locality, 1 plant specimen, 1 NIFDC reference material, 1 counterchecking specimen, Accession no. EF199848, EF199860	CACATCGCGTCACCCCATCCAAA ATAATGTTGGATGTGGTGGATTG TGACCTCCTGTGTCTTGAGATGC AGTTGGTCGAAATTCGAGTCCG GAGCTTAGGACTTCACGACAAG TGGTGGTTGGATAACAACTCGC GTCATGTCGTGTGCCAAGCCTCC GTGTCTCCGTAGACCCTAAGGC GCGTGCTTTCCAACTCGTTCGTT GGGAAACCGTGCTACGACCG	223	5 sites: A27C, A109G, C143T, G153A, T189C	[1]
**Radix Arnebiae**	*Arnebia guttata* Bunge	Accession JX976805	CGCATCGCGTCACCCCATCCCAT GTAACTATGGATGTGGTGGATTG TGACCTCCTGTGTCTTGAGATGC AGTTGGTCAAAATTTGAGTCTG GAGCTGAGGACTTCACGTCAAG TGGTGGTTGGATAACAACTCGC	221	-	[1,3]

			GTCCTGTCGTGTGCCAAACCTCC ATTCCTCTGTGGACCCTAAGGCG TGTTTTTTTCCAACTCTTGTGTT GGGAAATCTTGCTACGA			
**Radix Lithospermi**	*Lithospermum erythrorhizon* Sieb. Et Zucc	Plant specimens	CGCATCGCGTCACCCATCCCCAA CTCTCGTGGATGTGGTGGATTGT GACCTCCTGTGCCTTGCGGTGC AGTTGGTCGAAATTCGAGTTCG GAGTTAAGGACTTCACGACAAG TGGTGGTTGGACGACCAACTCG CGTCATGTCGTGTGCCAAACCTT CGTGGCTCTCAAGACCCTAAGG CACGTGCTACCCGACTCTTGGGT CGGAAAACTCGTGCTACGACCG	224	-	[1]

References

1. Chen, S.L. *Standard DNA barcodes of Chinese Materia Medica in Chinese Pharmacopoeia*, 1st ed.; China Science Publishing & Media Ltd.: Beijing, China, 2015; pp. 168, 287, 446, 492.
2. Xie, L.; Wen, J.; Li, L.Q. Phylogenetic analyses of Clematis (Ranunculaceae) based on sequences of nuclear ribosomal ITS and three plastid regions. *Syst. Bot*. **2011**, *36*, 907–921.
3. Huang, J.F.; Zhang, M.L.; Cohen, J.I. Phylogenetic analysis of *Lappula Moench* (Boraginaceae) based on molecular and morphological data. *Plant. Syst. Evol.* **2013**, *299*, 913–926.
